# Modifying the lipid content and composition of plant seeds: engineering the production of LC-PUFA

**DOI:** 10.1007/s00253-014-6217-2

**Published:** 2014-11-25

**Authors:** Noemi Ruiz-Lopez, Sarah Usher, Olga V. Sayanova, Johnathan A. Napier, Richard P. Haslam

**Affiliations:** Department of Biological Chemistry, Rothamsted Research, Harpenden, Herts AL5 2JQ UK

**Keywords:** Omega-3 fatty acids, Polyunsaturated fatty acid, Triacylglycerol, Transgenic plant, Oilseed, *Camelina sativa*

## Abstract

Omega-3 fatty acids are characterized by a double bond at the third carbon atom from the end of the carbon chain. Latterly, long chain polyunsaturated omega-3 fatty acids such as eicosapentaenoic acid (EPA; 20:5Δ5,8,11,14,17) and docosahexanoic acid (DHA; 22:6 Δ4,7,10,13,16,19), which typically only enter the human diet via the consumption of oily fish, have attracted much attention. The health benefits of the omega-3 LC-PUFAs EPA and DHA are now well established. Given the desire for a sustainable supply of omega-LC-PUFA, efforts have focused on enhancing the composition of vegetable oils to include these important fatty acids. Specifically, EPA and DHA have been the focus of much study, with the ultimate goal of producing a terrestrial plant-based source of these so-called fish oils. Over the last decade, many genes encoding the primary LC-PUFA biosynthetic activities have been identified and characterized. This has allowed the reconstitution of the LC-PUFA biosynthetic pathway in oilseed crops, producing transgenic plants engineered to accumulate omega-3 LC-PUFA to levels similar to that found in fish oil. In this review, we will describe the most recent developments in this field and the challenges of overwriting endogenous seed lipid metabolism to maximize the accumulation of these important fatty acids.

## Introduction

Lipids (oils and fatty acids) are indispensable for the growth and survival of all organisms. They are important structural components of membranes and they also play a crucial role in energy storage and signaling. Furthermore, several polyunsaturated fatty acids (FA) can act as metabolic precursors for eicosanoids. Natural sources of lipids include plants, animals, and microorganisms. Oils from plants (e.g., olive oil, sunflower oil) and animals (e.g., butter, lard) have always played a vital role in human life, providing multiple sources of food and fuel; recognized mostly for their applications in nutrition, but also their role as raw material in industrial processes. Currently, plant oils account for the majority of the natural oils and fats in the world, about 150 million metric tons of vegetable oils are consumed annually and this number is expected to increase in the future (OECD_FAO Agricultural Outlook 2012–2021). Plant oils are relatively inexpensive and are commonly considered to be healthier than animal fats, as they contain relatively high amounts of unsaturated fatty acids. However, over recent years, there has been increased interest in some specific animal oils, namely the long-chain polyunsaturated fatty acids (LC-PUFAs), found in fish oil. LC-PUFAs are vital constituents of human metabolism and have a recognized role in human health. Thus, the nutritional value of omega-3 LC-PUFAs is now widely appreciated. Plant oils are rich in C18 FA, including the essential FA linoleic acid (18:2Δ9,12 *n*-6; LA) and α-linolenic acid (18:3Δ9,12,15 *n*-3; ALA), but are devoid of LC-PUFAs, such as arachidonic acid (20:4Δ5,8,11,14, *n*-6; ARA), eicosapentaenoic acid (20:5Δ5,8,11,14,17 *n*-3; EPA) and docosahexaenoic acid (22:6Δ4,7,10,13,16,19 *n*-3; DHA), which typically only enter the human diet as oily fish. Although humans can synthesis both EPA and DHA (approximately 8 % of dietary ALA is converted to EPA and 0–4 % is converted to DHA), the supply does not meet nutritional demands. Equally, the decline in global fish stocks is also well established and compounded by the use of fish oils in aquaculture. For several years, we and others have suggested that transgenic oilseeds metabolically engineered with the heterologous capacity to synthesize omega-3 LC-PUFAs represent a novel (terrestrial) production system, which could substitute for some or all of the applications currently using fish oils.

## General principles of oilseed engineering

Success in agriculture has always depended on innovation; inventive approaches drive crop improvement, the selection of plants with desirable traits, and their successful propagation. In recent years, a better understanding of lipid assembly and storage, improved molecular genetics, and plant biotechnology have set the stage for a real breakthrough in the manipulation of oil crops for sustainable development. Specifically, genetic engineering based on recombinant DNA technology has now enabled the production of seed oils with a predictable FA composition; the incorporation of characteristics that are impossible to achieve by traditional breeding techniques.

Metabolic engineering of seed oil metabolism can be achieved via a wide range of methods. The most commonly used approaches are infection with *Agrobacterium tumefaciens* and biolistic gene transfer. *A. tumefaciens* is a widespread naturally occurring Gram-negative soil bacterium that causes crown gall, and has the ability to transfer a portion of DNA (transferred DNA or T-DNA) from its tumor-inducing (Ti) plasmid to plant cells (Gelvin 2003). The T-DNA contains the genes for inducing tumor formation and opine biosynthesis. In the laboratory, *Agrobacterium* bacterial T-DNA genes have been replaced by genes of interest. *Agrobacterium* is capable of transforming a range of dicotyledonous plant species; however, monocots are less responsive towards this method. The principle advantage of *Agrobacterium*-mediated gene transfer is the high occurrence of single-copy T-DNA integration with a relatively stable high level of transgene expression. In methods based on biolistics (or microprojectile bombardment), transformation is achieved by coating gold or tungsten microparticles with the desired DNA, and accelerating them using high pressure gas such that they are able to penetrate the cell (wall and membrane) and enter the plant nucleus. This method is routinely used for the transformation of monocots, cereals, legumes, and microalgae; species that are typically recalcitrant to *Agrobacterium*-mediated transformation (Sparks and Jones 2004). Bombardment offers a relatively high efficiency, the ability of delivering DNA without vector backbone, and also the opportunity to deliver DNA directly to organelle genomes. However, there can be substantial variation between experiments (due to differences in bombardment conditions), integrations at multiple loci, frequent truncations of the transgene and substantial damage to bombarded tissue, lowering regeneration capacity.

Several methods have been developed to overcome the limitations of *Agrobacterium*-mediated and particle bombardment gene delivery. One of them is a method of transformation using cell-penetrating peptides (CPPs). Chugh et al. (2009) demonstrated that the Tat monomer (Tat) and its dimer (Tat2) belong to the CPP family and are able to deliver DNA into plant cells. So far, this method has been used to deliver the transgene into microspores, but somatic cell targeting is still under development. This method has also been used in combination with an artificially produced T-DNA complex, allowing the production of transgenic triticale plants with great efficiency (Ziemienowicz et al. 2012). Latterly, efforts to improve crop performance have benefited from the arrival of a number of exciting techniques including mutagenesis and genome editing using Zinc finger nucleases (ZFN-1, ZFN-2, and ZFN-3); transcription activator-like effector nucleases (TALENs); oligonucleotide-directed mutagenesis (ODM); and gene inactivation using the bacterial CRISPR/Cas system (Wang et al. 2013; Balter et al. 2014; Lozano-Juste and Cutler 2014). Many of these approaches remain to be tested for their efficacy in manipulating seed oil assembly.

Oilseed crops and their different cultivars can have a highly variable fatty acid content and composition in their seeds with no significant differences in plant physiology. Therefore, the manipulation of oil content or/and composition should not dramatically alter plant physiology. Over the last two decades, there has been a substantial research effort to decipher the genes encoding the most important enzymes involved in FA and triacylglycerol (TAG) biosynthesis (for reviews, see Ruiz-Lopez et al. 2012a; Haslam et al. 2013; Li-Beisson et al. 2013; Napier et al. 2014), but further successful improvement of oil crops requires a good understanding of the endogenous biochemical processes that underpin seed oil assembly (Bates et al. 2013). With steady progress in genomics, proteomics, metabolomics, lipidomics, and genetic engineering as well as an increased number of genomes sequenced and annotated, the modification of oilseed crops becomes a more straightforward practice.

## Long chain polyunsaturated fatty acids

LC-PUFAs are composed of a long hydrocarbon chain (consisting of 20 or more carbon atoms) and a terminal carboxylate group containing more than two double bonds in their backbone. They are classified according to the position of the first double bond, as counted from the methyl terminus, which is denoted in the form (*n*-*x*), where *n* is the chain length of the fatty acid and *x* is the number of carbon atoms from the last double bond. Thus linoleate (LA) and α-linolenate (ALA) are 18:2(*n*-6) and 18:3(*n*-3), respectively (18:2ω6 and 18:3ω3 in the older literature). Double bonds in PUFAs may also be counted from the carboxylate group and are then represented by the symbol Δ. In Table 1, examples of several *n*-3 PUFAs are listed. Mammals lack the ability to introduce double bonds in FAs beyond carbon 9 and 10. Linoleic acid and α-linolenic acid found in plant oils are therefore an essential human dietary requirement (Hansen and Burr 1946) due to the lack of Δ-12- and Δ-15 desaturase activity. LA and ALA are precursors for the synthesis of LC-PUFAs. Today omega-3 LC-PUFAs such as EPA and DHA are particularly lacking in modern diets, leading to sub-optimal organ function and an increased risk of disease. A situation that, together with the fact that humans synthesize a limited amount of these PUFAs, has led many experts to recommend increased omega-3 LC-PUFA intake and make observable health benefits (FAO 2010).

**Table 1:** Lists of most common omega-3 and omega-6 fatty acids and their accumulation in microalgae and oilseed crops

Common name	Systematic name	Synonyms	Algae	Oilseeds
OMEGA3				
α-Linolenic acid (ALA)	9*Z*,12*Z*,15*Z*-octadecatrienoic acid	18:3*n*-3; 18:3 ^Δ9,12, 15^	√	√
Stearidonic acid (SDA)	6*Z*,9*Z*,12*Z*,15*Z*-octadecatetraenoic acid	18:4*n*-3; 18:4 ^Δ6,9,12, 15^	√	√^a^
Eicosatrienoic acid (ETE)	11*Z*, 14*Z*, 17*Z*-eicosatrienoic acid	20:3*n*-3; 20:3 ^Δ11,14,17^		
Eicosatetraenoic acid (ETA)	8*Z*,11*Z*,14*Z*,17*Z*-eicosatetraenoic acid	20:4*n*-3; 20:4 ^Δ8,11,14,17^	√	
Eicosapentaenoic acid (EPA)	5*Z*,8*Z*,11*Z*,14*Z*,17*Z*-eicosapentaenoic acid	20:5*n*-3; 20:5 ^Δ5,8,11,14,17^	√	
		22:5*n*-3;	√	
Docosapentaenoic acid (DPA)	7*Z*,10*Z*,13*Z*,16*Z*,19*Z*-docosapentaenoic acid	22:5 ^Δ7,10,13,16,19^		
Docosahexaenoic acid (DHA)	4*Z*,7*Z*,10*Z*,13*Z*,16*Z*,19*Z*-docosahexaenoic acid	22:6*n*-3; 22:6^Δ4,7,10,13,16,19^	√	
OMEGA6				
Linoleic acid (LA)	9*Z*,12*Z*-octadecadienoic acid	18:2*n*-6; 18:2 ^Δ9,12^	√	√
γ-Linolenic acid (GLA)	6*Z*,9*Z*,12*Z*-octadecatrienoic acid	18:3*n*-6; 18:3 ^Δ6,9,12^	√	√^a^
Dihomo-γ-linolenic acid (DGLA)	8*Z*,11*Z*,14*Z*-eicosatrienoic acid	20:3*n*-6; 20:3 ^Δ8,11,14^	√	
Arachidonic acid (ARA)	5*Z*,8*Z*,11*Z*,14*Z*-eicosatetraenoic acid	20:4*n*-6; 20:4 ^Δ5,8,11,14^	√	
Adrenic acid (DTA)	7*Z*,10*Z*,13*Z*,16*Z*-docosatetraenoic acid	22:4*n*-6; 20:4 ^Δ7,10,13,16^		
Docosapentaenoic acid (DPA*n*-6)	4*Z*,7*Z*,10*Z*,13*Z*,16*Z*-docosapentaenoic acid	22:5*n*-6; 20:4 ^Δ4,7,10,13,16^		

^a^Some Boraginaceae such as *Echium plantagineum* contain SDA, although this species is not generally recognized as an oilseed crop species. GLA is found in a few seed oils, and those of evening primrose, borage, and blackcurrant have some commercial importance

## Health aspects of *n-*3 LC-PUFAs

**Fig. 1:**
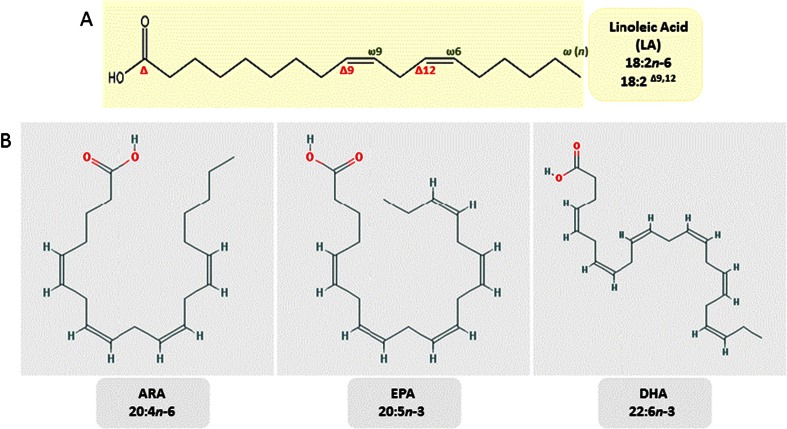
**a** Chemical structure of linoleic acid (LA, 18:2Δ9,12), consisting of a chain of 18 carbons with two double bonds on carbons Δ9,12, also named *n*-6,9. This nomenclature is taken from the location of the first double bond, counted from the carboxylic (Δ-) carbon or counted from the methyl (*n*) end. **b** Schematic representation of the 2D structures of arachidonic acid (ARA, 20:4*n*-6), eicosapentaenoic acid (EPA, 20:5*n*-3) and docosahexaenoic acid (DHA, 22:6*n*-3)

The position of the double bond in a fatty acid strongly affects the properties of its derivatives. For instance, eicosanoids derived from the *n*-6 LC-PUFA arachidonic acid have strong inflammatory properties, whereas those produced from *n*-3 LC-PUFA, e.g., eicosapentaenoic acid, are anti-inflammatory (Gill and Valivety 1997; Calder 2014). For reference, the chemical structures of DHA and EPA are shown in Fig. 1. The biological functions of omega-3 LC-PUFA (especially EPA and DHA) are now well established (Schmitz and Ecker 2007; Saravanan et al. 2010). DHA is an essential component of the lipids in cell membranes where it clearly exerts a structural and functional role, e.g., modifying membrane phospholipid composition, for instance, it accounts for over 60 % of the total FAs in the rod outer segment in the retina (Giusto et al. 2000). DHA also modulates properties of the hydrophobic core of the membrane bilayer, directly interacting with membrane proteins and is involved in lipid raft formation (Innis 2007). Furthermore, DHA is regarded to be essential for the proper visual and neurological development of infants (Crawford et al. 1997; Das and Fams 2003). Both EPA and DHA give rise to anti-inflammatory and inflammation resolving mediators called resolvins, protectins, and maresins; promoting the resolution of the inflammatory response back to a noninflamed state.

The protective effect of EPA and DHA in cardiovascular disease has been well documented in humans (Kromann and Green 1980; Kang and Leaf 1996; Nordøy et al. 2001; Balk et al. 2007). One of the main effects of dietary *n*-3 PUFA is to reduce elevated plasma TAG (Yokoyama et al. 2007). The hypotriglyceridemic effect of *n*-3 PUFA is believed to be due to their potent enhancement of lipolysis through activation of peroxisome proliferator-activated receptors (Nagao and Yanagita 2008). Additionally, long-chain PUFA are viewed as potent modulators of inflammation. Most of the mediators formed from EPA and DHA (leukotrienes, resolvins, and neuroprotectin/protectin D1) are anti-inflammatory, whereas those formed from the *n*-6 PUFA, arachidonic acid are mostly pro-inflammatory (Schmitz and Ecker 2007). Oxidative stress results from an imbalance between formation and degradation of pro-oxidants or decreased cellular antioxidant protection mechanisms, and may result in increased cell damage and apoptosis. DHA administration exerts antioxidant activity as shown by increasing glutathione reductase activity and decreasing accumulation of lipid peroxide and reactive oxygen species in the cortex and hippocampus of AD model rats (Guillot et al. 2008; Hashimoto et al. 2002, 2008). However, the few human studies examining the impact of DHA supplementation on oxidative stress have yielded inconsistent results. Finally, it has also been established that omega-3 LC-PUFA have some positive effects on diseases such as hypertension, arthritis, arteriosclerosis, and thrombosis (Horrocks and Yeo 1999), and are involved in the protection, might possibly even enhance, the effect of medical treatment diseases such as Alzheimer’s, multiple sclerosis, and cancer. At present, most FAs entering the human diet originate from plant oils and belong to the *n*-6 group. It is probable that humans evolved on a diet with a ratio of omega-6 to omega-3 essential fatty acids (EFA) of approximately 1 whereas in Western diets the ratio is now 15/1–16.7/1. In order to restore the fatty acid balance, generally seen as optimal for human health, an increase in *n*-3 (LC)-PUFA consumption and a reduction in *n*-6 PUFAs is needed. Collectively, there is a demand for a sustainable solution to EPA and DHA supply.

## Current sources of DHA

Currently marine fish (oily fish species, such as herring, mackerel, sardine, and salmon) and seafood are the primary dietary source of these beneficial omega-3 LC-PUFAs (Gunstone 1996), including DHA and EPA. Fish concentrate them by ingesting marine microalgae that are able to synthesize PUFAs de novo. Besides microalgae, some lower plants, fungi, bacteria, and moss can also synthesize LC-PUFA; however, crucially no higher plants can produce LC-PUFAs. The quality of fish oil is variable and depends on fish species, season, and location of catching sites. Fish oils cannot meet the ever increasing global demand for *n*-3 LC-PUFAs because worldwide fish stocks are declining. Additionally, the contamination of the fish oil by environmental pollution of marine ecosystems has become pervasive and global problem (Domingo et al. 2007; Tocher 2009). Furthermore, as marine fish oil is a complex mixture of FAs with varying lengths and degrees of unsaturation, expensive DHA purification may be required. Notwithstanding these issues, fish oil is sometimes perceived to have an unpleasant smell and taste. Krill oil has also been suggested as an alternative to fish and microalgal oils to fulfill the dietary demand for EPA and DHA. The EPA and DHA in fish are mostly in the form of TAG, whereas krill oil has TAG, but also phospholipids and nonesterified fatty acid forms of these FAs. Some studies have suggested that the EPA and DHA in krill oil is more bioavailable, however this may in fact be due to study design limitations (Salem and Kuratko 2014). The demands for omega-3 LC-PUFA continues to rise, due to a rapid increase in aquaculture and applications in food and pharmacy. It is therefore expected that the production of LC-PUFAs from current sources will become inadequate for supplying the expanding market in the near future. Collectively, these circumstances have led to an increasing interest in the search for alternative sustainable sources of omega-3 LC-PUFAs. Presently, there are two potential alternatives to fish oils: EPA and DHA from metabolically engineered plant oilseeds and microbial single cell oils (see Adarme-Vega et al. 2014).

## LC-PUFA biosynthetic pathways

The primary producers of LC-PUFA mostly consist of marine bacteria, fungi, protists, and microalgae. Within these organisms, two separate biochemical biosynthetic pathways have been identified: (1) the aerobic desaturase/elongase pathway (Sayanova and Napier 2004); and (2) the anaerobic polyketide synthase (PKS) pathway (Metz et al. 2001). A large amount of research effort has been committed to characterizing these two biosynthetic pathways.

### Aerobic pathway

EPA, DHA, and other LC-PUFAs are traditionally considered to be products of alternating desaturation and elongation steps acting on long-chain (C18) polyunsaturated substrates; therefore, two distinct types of primary biosynthetic activities (desaturases and elongases) are required. To date, several sets of genes encoding these enzymes have been isolated and identified from a wide range of prokaryotic and eukaryotic species (Pereira et al. 2003; Hashimoto et al. 2008). The first step is a desaturation which is catalyzed by a front-end desaturase, which introduces a double bond between the pre-existing double bond and the carboxyl end of the fatty acid substrate, as opposed to methyl-end desaturases that insert a double bond between the pre-existing double bond and the methyl end (Meesapyodsuk and Qiu 2012). Desaturases involved in LC-PUFA synthesis normally include three highly conserved histidine-rich sequences (His boxes) with the general motifs H-X[3,4]H, H-X[2,3]H-H, and H/Q-X[2,3]H-H (Shanklin and Cahoon 1998; Sperling et al. 2003). Front-end desaturases from eukaryotes (Mitchell and Martin 1995; Sayanova et al. 2001; Napier et al. 1999, 2003) have a number of unique characteristics; namely, they contain an N-terminal cytochrome b5 fusion domain (Sayanova et al. 1997) that presumably acts as electron receptor for the desaturation reaction and they have a histidine/glutamine substitution in the third His box. To date, these front-end desaturases have been mostly identified in animals and microorganisms. The majority of the microsomal desaturases from lower eukaryotes use glycerolipid-linked substrates, in particular FAs esterified to the *sn*-2 position of glycerolipids. This is in contrast to animals and microalgae, in which the substrates for these enzyme activities are thought to be acyl-CoAs (Stymne and Appelqvist 1978; Griffiths et al. 1988; Jackson et al. 1998; Domergue et al. 2005). Methyl-end desaturases lacking the cytochrome b5 domain, such as membrane-bound *n*-3, Δ12-, and Δ15-desaturases, also commonly occur in plants, algae, and some fungi.

The second step in LC-PUFA biosynthesis is microsomal FA elongation by two carbons, catalyzed by an elongation complex consisting of four discrete subunits: a β-ketoacyl-CoA synthase (KCS, the condensing enzyme), a ketoacyl-CoA reductase, a hydroxyl acyl-CoA dehydratase, and an enoyl-CoA reductase (Fehling et al. 1992). However, the heterologous expression of just the initial condensing enzyme is capable of reconstituting the heterologous elongating activity (Millar and Kunst 1997; Paul et al. 2006) and for this reason KCS are often referred to as “elongases”. It is widely accepted that the substrate specificity of the elongation complex (in terms of chain length and degree of unsaturation) is primarily determined by the KCS, rather than the other three components (Millar and Kunst 1997). Furthermore, the turnover of KCS activity is considered to be the rate limiting step for the elongase complex. In terms of classification, there are two groups of KCS which encompasses all those identified from plants, animals, and eukaryotic microorganisms: the FAE1-like plant-specific KCS activities involved in the biosynthesis of saturated and monounsaturated FAs with C18 to C22+ chain length (James et al. 1995; Leonard et al. 2004) and the ELO-like enzymes (some of which are involved in LC-PUFA biosynthesis). Several ELO-like sequences have been identified and heterologous studies have demonstrated that they can elongate PUFAs with chain lengths of 18-carbons or more (Dittrich et al. 1998; Parker-Barnes et al. 2000; Meyer et al. 2004; Pereira et al. 2004). In contrast to microsomal desaturation, all the microsomal elongation described so far, both ELO-type and FAE1-like, exclusively uses acyl-CoAs as substrates (Jakobsson et al. 2006).

**Fig. 2:**
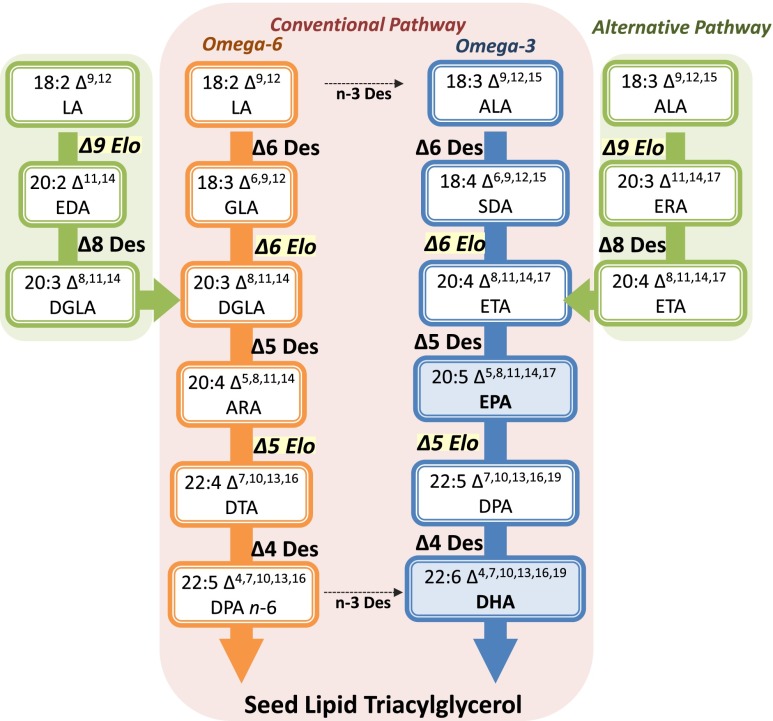
A schematic representation of the conventional LC-PUFA Δ6-biosynthetic pathway from linoleic and α-linolenic acid precursors. The alternative Δ8-pathway utilizes a Δ9-elongase and Δ8-desaturase to produce DGLA and ETA, respectively

The synthesis of LC-PUFAs in plants begins with the production of FAs by the multi-subunit fatty acid synthase (FAS) complex in the plastid (Harwood 1988; Somerville and Browse 1991). Stearic acid (18:0), the final product of FAS, is typically desaturated to oleic acid (OA, 18:1Δ9) by a stearoyl-acyl carrier protein (ACP) Δ9-desaturase. Almost all higher plants, some cyanobacteria, and many *n*-3 LC-PUFA producing microorganisms have Δ12-, and Δ15-/*n*-3 desaturases to convert OA into linoleic acid and α-linolenic acid while animals, including humans, are deficient in them. Additional conversion of LA and ALA to *n*-3 LC-PUFAs needs the introduction of appropriate front-end desaturases, and C18/C20-PUFA-specific elongases (Fig. 2). There are two separate and converging pathways in LC-PUFA synthesizing organisms: the conventional pathway (in most *n*-3 LC-PUFA synthesizing eukaryotic organisms) and the alternative pathway found in the protists such as *Euglena gracilis*, *Tetrahymena pyroformis*, *Acanthamoeba* spp., and some species of microalgae (Lees and Korn 1966; Ulsamer et al. 1969; Wallis and Browse 1999). In the conventional pathway, biosynthesis of LC-PUFAs starts with the Δ6-desaturation of LA (*n*-6) and ALA (*n*-3), generating γ-linolenic acid (GLA, 18:3 Δ6,9,12) and stearidonic acid (SDA, 18:4 Δ6,9,12,15), respectively. These Δ6-desaturated FAs are then elongated to yield dihomo-γ-linolenic acid (DGLA, 20:3 Δ8,11,14) and eicosatetraenoic acid (ETA, 20:4 Δ8,11,14,17), respectively. Finally, a Δ5-desaturase carries out one more desaturation to produce ARA (*n*-6) and EPA (*n*-3), respectively (Michaelson et al. 1998). In the alternative pathway, biosynthesis of LC-PUFAs is initiated by the Δ9-elongation of LA and ALA generating eicosadienoic acid (EDA, 20:2 Δ11,14) and eicosatrienoic acid (ERA, 20:3Δ 11,14,17), respectively (Wallis and Browse 1999; Qi et al. 2002; Li et al. 2011). These Δ9-elongated FAs are then desaturated by a Δ8-desaturase to yield DGLA and ETA, respectively. Subsequently, these FAs can then be Δ5-desaturated to form ARA and EPA, respectively. The two *n*-6 and *n*-3 pathways can be interconnected by omega-3 desaturases, which convert *n*-6 FAs into their *n*-3 counterparts. From this point, the biosynthesis of DHA may follow two different routes: either the “linear” or Sprecher pathway. In DHA-producing microbes, DHA is synthesized by the “linear” pathway in which a specific Δ5-elongation of EPA by a Δ5/ C20-elongase yields docosapentaenoic acid (DPA, 22:5 Δ7,10,13,16,19), followed by a Δ 4-desaturation to form DHA. Several genes encoding Δ5-elongating or Δ4-desaturation activities have been isolated and functionally characterized (Pereira et al. 2004; Qiu et al. 2001; Meyer et al. 2003). Conversely, mammals are thought to synthesize DHA by the Sprecher pathway in which EPA is twice elongated, yielding 24:5 *n*-3, desaturated to 24:6 *n*-3, and finally shortened to DHA via beta oxidation, a route independent of Δ4-desaturation (Sprecher et al. 1999; Burdge 2006).

### Anaerobic pathway

The anaerobic or polyketide synthase (PKS) pathway represents an alternative route to EPA and DHA; identified in both prokaryotic and eukaryotic microorganisms (Metz et al. 2001). First described in the marine bacteria *Shewanella pneumatophori* strain SCRC-2378 (Yazawa 1996), the complex consists of eight different PKS protein domains, including malonyl-CoA:ACP acyltransferase (MAT), acyl carrier protein (ACP), 3-ketoacyl synthase (KS), 3-ketoacyl-ACP reductase (KR), acyltransferase (AT), chain length factor (CLF), enoyl reductase (ER), and a 3-hydroxyacyl-ACP dehydratase/isomerase (DH/IS). Although the precise mechanism by which some marine bacteria and low eukaryotes synthesize large amounts of EPA or DHA within the cell remains uncertain, a hypothetical pathway has been proposed (Wallis et al. 2002; Napier 2002). Duplicating the aerobic pathway, biosynthesis via PKS also involves several rounds of reduction, dehydration, reduction, and condensation; in each cycle the fatty acyl chain is extended by a two-carbon unit while a double bond is introduced every three carbons along the acyl chain (Kaulmann and Hertweck 2002; Cao et al. 2012). Several rounds of sequential reactions are repeated and result in the formation of LC-PUFA with 20 or 22 carbons and several double bonds that are methylene-interrupted, i.e., EPA or DHA. Unlike the aerobic pathway, the anaerobic PKS pathway inserts double bonds most likely through the action of a FabA-like dehydratase/isomerase module during the iterative extension of the fatty acyl chain and utilizes acetyl-CoA as the putative primer molecule to synthesize EPA or DHA. Interestingly, these two distinct LC-PUFA biosynthetic pathways (aerobic and anaerobic) have been found to coexist in the same microbial species such as *Schizochytrium* and *Thraustochytrium aureum* (Metz et al. 2001; Qiu et al. 2001).

## Engineering LC-PUFA in higher plants

Although it has been some years since the successful reconstitution of the omega-3 LC-PUFA biosynthetic pathway in plants using multiple desaturases and elongases, only recently have seed oils been engineered to produce EPA and/or DHA at levels matching fish oils (Ruiz-Lopez et al. 2014). Today, the stable transformation of different oilseeds crops with multiple genes is not the technical barrier; rather issues around endogenous acyl-exchange have caused bottlenecks for engineering plants to produce high levels of *n*-3 LC-PUFAs. To successfully convert native plant FAs such as LA and ALA to LC-PUFAs such as EPA and DHA in seeds require the coordinated expression of multiple genes, as a minimum of three sequential nonnative enzymatic reactions are required (e.g., two desaturations and an acyl-CoA elongation; see Fig. 1).

### Substrate limitations on LC-PUFA synthesis

The first bottleneck (substrate dichotomy) was identified as researchers endeavored to reconstruct either partially or entirely the LC-PUFA biosynthetic pathways in the yeast *Saccharomyces cerevisiae*. In this in vivo study, six Δ6-desaturases, five Δ5-desaturases, and two Δ12-desaturases genes from different organisms were expressed in *S. cerevisiae* either separately or in combination. Domergue et al. (2003) demonstrated that unlike mammalian desaturases that use acyl-CoAs as substrates, the front-end desaturases used in the study showed preferential specificity for acyl groups esterified at the *sn*-2 position of phospholipids, e.g., phosphatidylcholine (PC).

In plants, the seed-specific expression in transgenic tobacco (*Nicotiana tabacum*) and linseed (*Linum usitatissimum*) of cDNAs encoding fatty acyl-desaturases and elongases, resulted in the very high accumulation of Δ6-desaturated C18 FAs and a low accumulation of ARA and EPA (Table 2; Abbadi et al. 2004). Moreover, detailed lipid analyses of developing seeds revealed that after desaturation on PC, Δ6-desaturated products were immediately channeled to the triacylglycerols and effectively bypassed the acyl-CoA pool. Therefore, the lack of Δ6-desaturated acyl-CoA substrates in the acyl-CoA pool limited the synthesis of elongated C20 FAs, and rendered the production of LC-PUFA in transgenic oilseeds unsuccessful. This bottleneck was described as a “substrate dichotomy” (Napier 2007), and it is the consequence of desaturase and elongase activities requiring different substrates, namely phospholipid-linked substrates for desaturases and acyl-CoA for elongases. Qi et al. (2004) reconstituted the alternative Δ8-desaturase pathway in *Arabidopsis* plants with genes expressing a Δ9-elongase, a Δ8-desaturase, and Δ5-desaturase under the control of the constitutive 35S promoter. The accumulation of 6.6 % ARA and 3.0 % EPA in total lipids of leaf tissues represented a “proof-of-concept” demonstration for the plant synthesis of LC-PUFAs and for the functionality of the alternative pathway in plants. However, further detailed analyses of leaf lipids indicated an inefficient transfer of these nonnative FAs from the acyl-CoA pool into extraplastidial phospholipids (Fraser et al. 2004; Sayanova et al. 2006).

**Table 2:** Comparison of published transgenic lines producing LC-PUFA and biosynthetic intermediates

References	Plant species	Tissue	GLA	SDA	DGLA	ARA	ETA	EPA	DPA	DHA
Conventional pathway										
Abbadi et al. 2004	*N. tabacum*	Seed	29.3	–	1.8	1.5	–	–	–	–
	*L. usitatissimum*	Seed	16.8	11.4	1.2	1.0	0.9	0.8	–	–
Kinney et al. 2004	*G. max*	Embryo	22.7	3.1	4.0	0.4	3.3	13.3	0.9	–
	*G. max*	Embryo	2.7	3.6	3.1	2.5	2.1	5.2	1.0	3.3
	*G. max*	Seed	11.7	1.1	10.1	2.2	2.4	19.6	0.8	–
Wu et al. 2005	*B. juncea*	Seed	27.3	2.2	1.9	4.0	1.1	8.1	0.1	0.2
Ruiz-López et al. 2009	*L. usitatissimum*	Seed	>0.5	11.8	–	–	–	–	–	–
Cheng et al. 2010	*B. carinata*	Seed	26.9	5.4	2.2	5.7	2.5	20.4	4.0	–
Ruiz-Lopez et al. 2012a	*A. thaliana*	Seed	17.7	8.1	0.3	2.1	nd	4.1	–	–
Alternative pathway										
Qi et al. 2004	*A. thaliana*	Leaf	–	–	1.3	6.6	1.2	3.0	–	–
Using Acyl-CoA desaturases										
Robert et al. 2005	*A. thaliana*	Seed	0.6	1.8	1.9	1.6	0.4	3.2	0.1	–
	*A. thaliana*	Seed	0.4	1.5	1.5	1.0	0.8	2.4	0.1	0.5
Hoffmann et al. 2008	*A. thaliana*	S seed	>0.5	>0.1	0.8	0.1	0.9	0.05	–	–
Petrie et al. 2010a, b	*N. benthamiana*	Leaves	2.1	1.5	–	0.6	0.6	10.7	0.3	–
Ruiz-Lopez et al. 2012b	*A. thaliana*	Seeds	1.7	0.8	0.2	6.2	nd	4.0	–	–
Petrie et al. 2012a	*A. thaliana*	T4 seeds	0.4	4.8	–	–	0.8	1.5	1.1	13.3
Ruiz-Lopez et al. 2013	*A. thaliana*	T3 seeds	1.9	1.1	1.5	3.2	0.9	13.2	–	–
	*A. thaliana*	T2 seeds	2.8	1.6	0.6	1.0	nd	3.4	1.1	2.5
Petrie et al. 2014	*C. sativa*	S seed	1.2	8.9	–	–	0.4	3.3	0.8	12.4
Mansour et al. 2014	*C. sativa*	Line (seeds)	–	3.3	–	–	0.4	0.2	1.1	4.2
Ruiz-Lopez et al. 2014	*C. sativa*	S seed	1.6	1.4	0.4	1.2	2.7	12.6	np	13.7
	*C. sativa*	Line (seeds)	2.7	2.2	0.9	2.2	3.5	11.3	np	7.7
PKS system										
Metz et al. 2006	*A. thaliana*	Seed	nd	nd	nd	nd	nd	nd	1.8	2.4

Note the tissues column describes the site of targeted gene expression and subsequent choice of material for analysis, i.e., seed (or *s seed* which refers to single seed analysis), embryo, leaf, line (where seed from a specific transgenic line was chosen for analysis) and T2 to T4 which refers to analysis of seed harvested from transgenic line generation 2 to 4

Resolving the substrate dichotomy problem led researchers to identify at least three acyl-CoA-dependent Δ6-desaturases from microalgae. Heterologous expression of the acyl-CoA Δ6-desaturase from *Ostreococcus tauri* in *S. cerevisiae* revealed very high desaturation activity with Δ6-regioselectivity, desaturating ALA to SDA with a conversion rate of 71–73 %, much higher than that of other lipid-linked Δ6-desaturases (Domergue et al. 2005). Moreover, short-time kinetic experiments showed that the desaturase product was detected in the acyl-CoA pool. In addition, using LA or ALA as exogenous substrate, both the ARA and EPA biosynthetic pathways were efficiently reconstituted in yeast by co-expressing OtD6 together with a moss Δ6-elongase and a microalgal Δ5-desaturase. Using this combination, the synthesis of relatively high yields of LC-PUFAs was achieved and they were associated with very high elongation efficiency of OtD6 desaturated intermediates (95 %), which bypasses the endogenous acyl-exchange between PC and the acyl-CoA pool. Sayanova et al. (2012) evaluated this activity in transgenic yeast, *Arabidopsis thaliana* and *Camelina sativa*, demonstrating that the use of acyl-CoA-dependent Δ6-desaturases almost completely abolished the accumulation of unwanted biosynthetic intermediates such as γ-linolenic acid in total seed lipids and that the expression of acyl-CoA Δ6-desaturases resulted in increased distribution of long-chain polyunsaturated FAs in the polar lipids of transgenic plants, reflecting a larger substrate pool available for acylation by enzymes of the Kennedy pathway. By contrast, MsD6, another acyl-CoA Δ6-desaturase isolated from the microalga *Mantoniella squamata*, only converted ALA into SDA (Hoffmann et al. 2008). The EPA biosynthetic pathway was also reconstituted by co-expressing the moss Δ6-elongase (PSE1; Zank et al. 2002) together with MsD6 and acyl-CoA Δ5-desaturase (MsD5). Transgenic seeds accumulated low levels (<0.5 %) of EPA, but lacked the accumulation of Δ6-desaturation products previously observed. A third acyl-CoA Δ6-desaturase was recently identified in the marine microalga *Micromonas pusilla*, and its heterologous expression in yeast also led to Δ6-desaturation of 71 % of exogenous ALA into SDA with no enrichment in the PC (Petrie et al. 2010a), indicating a strong preference for *n*-3 substrates.

Petrie et al. (2010b) went on to demonstrate how up to 26 % EPA could be produced in *N. benthamiana* leaf TAGs (10.7 % in total FAs) using this *M. pusilla* putative acyl-CoA-dependent Δ6-desaturase. These experiments make use of a very useful and exciting tool reported by Wood et al. (2009); a leaf-based transient expression was used to identify a set of omega-3 LC-PUFA biosynthetic genes with high enzyme activities and the desired substrate (acyl-CoA) preference. This new approach could certainly enhance our capacity to identify some optimal combinations of FA biosynthetic activities for the production of LC-PUFAs in plants, provided that any differences between leaf cells and developing cotyledon cells in seeds are accounted for. The utility of this approach has allowed for the rapid validation of seed-specific constructs, which would otherwise be dependent on stable transformation (Petrie et al. 2010b). With this knowledge, the authors were then able to assemble a large T-DNA construct for stable seed-specific transformation of *Arabidopsis*, and reported a high level of DHA (but not EPA) in seed oil (Petrie et al. 2012a) and separately yielded lines accumulating significant ARA (Petrie et al. 2012b).

In an attempt to identify the optimal combination of biosynthetic enzymes (desaturases and elongases) for LC-PUFA production in seeds, a systematic study was carried out evaluating 12 different constructs (combinations of three to seven transgenes) in *Arabidopsis* (Ruiz-Lopez et al. 2013). The authors evaluated the contribution of the different transgene enzyme activities, as well as the contribution of endogenous fatty acid metabolism. Successive iterations were then informed by lipidomic analysis; an approach that enabled a significant improvement on levels previously reported for the accumulation of EPA in *Arabidopsis* seeds and also facilitated the successful engineering of the high-value polyunsaturated fatty acid DHA to ten-fold higher levels. Collectively, these studies demonstrated that the accumulation of significant (meaning similar to that found in fish oils) levels of EPA and/or DHA is achievable in plants. More recently, Ruiz-Lopez et al. (2014) described two different iterations carrying a set of heterologous genes capable of efficiently directing synthesis of these FAs in the seed oil of the crop *C. sativa*, while simultaneously avoiding accumulation of undesirable intermediate FAs, and reporting the highest levels of C20+ omega-3 LC-PUFAs in a common oilseed crop. In the first iteration, seeds contained EPA levels of up to 31 % (mean 24 %) and in the second one, seeds accumulate up to 12 % EPA and 14 % DHA (mean 11 % EPA and 8 % DHA). These omega-3 LC-PUFA levels are equivalent to those in fish oils, and represent a sustainable, terrestrial source of these FAs. The engineering of oilseed crops has led to the accumulation of omega-3 LC-PUFAs at fish oil levels, demonstrating the efficacy of acyl-CoA desaturases over previously used lipid-linked desaturases.

### Triacylglycerol assembly and endogenous acyl-exchange

**Fig. 3:**
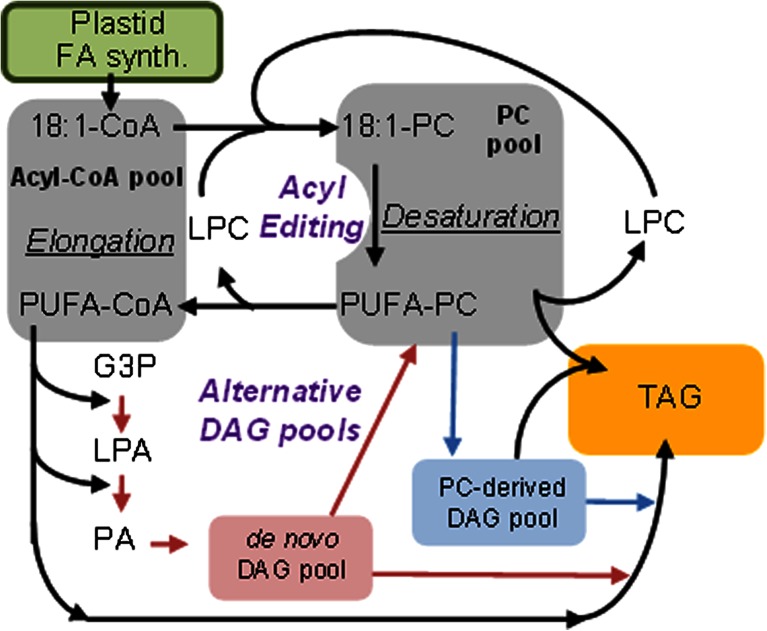
Overview of the major reactions involved in fatty acid and triacylglycerol synthesis (adapted with permission from Bates et al. 2013)

The de novo assembly of TAG from glycerol-3-phosphate (G3P) (also known as the Kennedy pathway) involves four enzymatic steps: first, two acylations of G3P by *sn*-1 glycerol-3-phosphate acyltransferase (GPAT) and lyso-phosphatidic acid acyltransferase (LPAAT), followed by phosphatidic acid phosphatase (PAP), and a third acylation by diacylglycerol acyltransferase (DGAT). However, TAG biosynthesis in seeds is not a simple linear pathway, but a complex network of reactions involving multiple subcellular compartments, parallel paths, cycles, and crossover with membrane lipid synthesis. The relative flux of FAs through different parts of the network affects the availability of the FAs for modification, e.g., desaturation and elongation (Fig. 3). FAs are synthesized with up to 18 carbons and one double bond in the plastid; subsequent Δ12(*n*-6)- and Δ15(*n*-3)-desaturations occur in the ER after incorporation of oleoyl groups as ester components into PC. In seeds engineered with new gene activities, additional desaturation and elongation continues in the acyl-CoA pool to produce LC-PUFA. The assembly of LC-PUFA in TAG then requires the coordinated flux of FA between the acyl-CoA pool and PC (Bates et al. 2013). Plants contain two main mechanisms for FA flux into and out of PC for desaturation: (1) Acyl editing, a PC deacylation and lyso-PC reacylation cycle that exchanges FAs with the acyl-CoA pool; (2) utilization of a PC-derived diacylglycerol (DAG) pool for TAG synthesis, rather than DAG synthesized de novo from acyl-CoA (Fig. 3). The exchange of FA from the PC pool to DAG producing TAG via phospholipid:diacylglycerol acyltransferase (PDAT) activity is unclear. PDATs from *Arabidopsis* and other plants have been shown to have high activity with PC containing unusual FA suggesting that PDAT may be involved in the removal of damaged or unusual FA from the membrane and sequestering them in TAG, a role which is potentially beneficial to the production of LC-PUFA in seed oil. The level of complexity is compounded in seeds by three other routes to DAG from PC; (1) phosphatidylcholine:diacylglycerol cholinephosphotransferase (PDCT), which exchanges phosphocholine between PC and DAG; (2) the reverse reaction of CDP-choline:diacylglycerol cholinephosphotransferase (CPT) and (3) lipase-based mechanism utilizing phospholipase C, or phospholipase D. Finally, rather than a circuitous route via PC and DAG, acyl-CoA can directly be incorporated into the *sn*-3 position of TAG via diacylglycerol acyltransferase (DGAT) activity. Plants are known to have multiple DGAT enzymes, which appear to have roles in the assembly of seed oil. Individually or collectively, each mechanism differently affects the availability of LC-PUFAs as substrates for TAG synthesis, and thus the composition of the seed oil. Moreover, the flux through the respective pathways of TAG synthesis appears to differ between plant species ranging from just a simple Kennedy pathway to a pathway where >90 % of the FAs within the seed fluxes through PC before incorporation into TAG (Bates and Browse 2012), therefore the suitability of different oilseed crops for metabolic engineering is species dependent and must be accommodated in any seed oil modification strategy.

## Conclusions and future outlook

After the initial successful introduction into plants of the primary biosynthetic enzymes for the production of LC-PUFA was demonstrated, the recent engineering of economically viable levels of EPA and DHA in the oilseed crop *Camelina* now represents a tangible success. Equally, the numerous engineering iterations that have arisen during this process have highlighted our incomplete appreciation of the pathways of fatty acid synthesis and lipid remodeling. The benefits of producing LC-PUFA in plants are clear: a sustainable and noncontaminated source of important fatty acids essential to human nutrition. Once a route for a LC-PUFA trait through performance field trials and regulatory approval is secured, the entry of transgenic LC-PUFA into the human food chain is possible. This might occur directly through products formulated with transgene-derived LC-PUFA, e.g., yogurt, margarine or indirectly, in animal feeds containing transgene-derived LC-PUFA, e.g., most obviously in farmed fish fed on a diet of modified terrestrial oils. Solving bottlenecks that limit the synthesis and accumulation of novel fatty acids has demonstrated the need for a more in-depth understanding of fatty acid metabolic pathways in seeds. Successful seed oil engineering necessitates a detailed understanding of the relative contributions of different enzymes specialized for TAG assembly in the native species and to possibly downregulate nonproductive or competing pathways. The desire to produce LC-PUFA has focused attention on fundamental metabolic processes and the application of new technology to characterize their interaction; further advances in our knowledge of plant lipid biochemistry will undoubtedly occur. It is clear that the future success of metabolic engineering of specialty oil traits will require greater predictability of genetic modifications on fatty acid and oil metabolism in seeds by use of techniques, such as mass spectrometry-based lipidomics. Ultimately, the challenging task of modifying seed oil remains the integration of a few transgene-derived activities into a greater number of endogenous metabolic processes.
